# Platelet mass index, systemic immune-inflammation index, and neutrophil-lymphocyte ratio as practical markers in childhood brucellosis

**DOI:** 10.1590/1984-0462/2025/43/2024123

**Published:** 2025-01-17

**Authors:** Hilal Koyuncu, Ayşe Tolunay Oflu, Ayşe Güngör, Ayşegül Bükülmez

**Affiliations:** a Afyonkarahisar Health Sciences University, Faculty of Medicine, Department of Child Health and Diseases, Afyonkarahisar, Turkey.

**Keywords:** Brucellosis, Inflammation, Platelet, Systemic, Brucelose, Inflamação, Plaquetária, Sistêmico

## Abstract

**Objective:**

Brucellosis is a multisystem infectious disease and may cause an increase in acute phase reactants. This study aimed to examine the platelet mass index (PMI), the neutrophil-lymphocyte ratio (NLR), and systemic immune-inflammation index (SII) in children with brucellosis and to determine their roles in focal involvement.

**Methods:**

This retrospective observational study included 69 patients with brucellosis and a control group of 69 healthy children. Inflammation markers, PMI, NLR, and SII were compared in brucellosis patients and the control group and in brucellosis patients with and without focal involvement.

**Results:**

Hemoglobin and platelet values were significantly lower in brucellosis cases compared to the control group (p<0.001), and C-reactive protein and ferritin were significantly higher (p<0.001). SII and PMI were significantly lower in the brucellosis patient group compared to the control group (p<0.001). In the comparing cases with and without focal involvement, no statistically significant difference was detected in hematological parameters and inflammation markers.

**Conclusions:**

This study showed that PMI, one of the new markers that can be calculated from complete blood count, may be useful in diagnosing childhood brucellosis. Similar results could not be reached for NLR and SII. New studies testing the diagnostic value of PMI, NLR, and SII in childhood brucellosis are needed.

## RESUMO

**Objetivo:**

A brucelose é uma doença infecciosa multissistêmica e pode causar aumento dos reagentes de fase aguda. Este estudo teve como objetivo examinar o índice de massa plaquetária (PMI, *platelet mass index*), a relação neutrófilos-linfócitos (NLR, *neutrophil-lymphocyte ratio*) e o índice de inflamação imunológica sistêmica (SII, *systemic immune-inflammation index*) em crianças com brucelose, e determinar seus papéis no envolvimento focal.

**Métodos:**

Este estudo observacional retrospectivo incluiu 69 pacientes com brucelose e um grupo controle de 69 crianças saudáveis. Marcadores de inflamação, PMI, NLR e SII foram comparados em pacientes com brucelose e grupo controle e em pacientes com brucelose com e sem envolvimento focal.

**Resultados:**

Os valores de hemoglobina e plaquetas foram significativamente menores nos casos de brucelose em comparação ao grupo controle (p<0,001), e proteína C-reativa e ferritina foram significativamente maiores (p<0,001). O SII e o PMI foram significativamente menores no grupo de pacientes com brucelose em comparação ao grupo controle (p<0,001). Na comparação dos casos com e sem envolvimento focal, não foi detectada diferença estatisticamente significativa nos parâmetros hematológicos e marcadores de inflamação.

**Conclusões:**

Este estudo mostrou que o PMI, um dos novos marcadores que podem ser calculados a partir do hemograma completo, pode ser útil no diagnóstico da brucelose infantil. Resultados semelhantes não puderam ser alcançados para NLR e SII. Novos estudos são necessários para testar o valor diagnóstico de PMI, NLR e SII na brucelose infantil.

## INTRODUCTION

Brucellosis is the most common zoonosis worldwide, caused by intracellular bacteria of the genus *Brucella*.^1^ More than 500,000 cases of brucellosis are reported yearly to the World Health Organization (WHO) from 100 countries, and most cases occur in the Mediterranean region.^2^ It is transmitted from infected animals to humans through direct contact or consumption of unpasteurized fresh milk and dairy products.^2^ The most common symptoms in pediatric brucellosis are fever, joint pain, chills, sweating, weakness, weight loss, abdominal pain, myalgia, and cases may present with multisystem involvement. It is confused with many other infectious and non-infectious diseases for its nonspecific clinical and laboratory features.^1^

The gold standard test for the diagnosis of brucellosis is the culture method. The 1/160 standard tube agglutination test (STAT), which is easier to perform, is the most common diagnostic tool.^2^ Laboratory findings such as leukocytosis/leukopenia, relative lymphocytopenia, anemia, thrombocytopenia, and elevation in acute phase reactants, which are frequently seen in patients, are findings that support the diagnosis.^3^ In several previous studies, inflammatory markers such as neutrophil-lymphocyte ratio (NLR), platelet-lymphocyte ratio (PLR), and neutrophil/monocyte ratio (NMR), which can be easily calculated from complete blood count, have also been investigated as diagnostic tools.^4^ The release of inflammatory cytokines causes several different changes in blood cells. As a result of these changes, many new inflammatory markers such as NLR, NMR, and systemic immune-inflammation index (SII) have been identified.^5^ Today, NLR is widely used in various pathological conditions as a reliable and easily obtainable marker of the immune response in a variety of infectious and non-infectious conditions.^6^ It has been reported that NLR increases in appendicitis and severe pneumonia in children,^7,8^ and SII is associated with poor prognosis in COVID-19 adult patients and increases in empyema and myocarditis in children.^9-11^ It is known that platelets also play an active role in inflammatory conditions.^12^ Researches have shown that the functions of platelets are better defined by the platelet mass index (PMI) described in recent years. It was found to be significant as a prognostic factor in sepsis in children.^13^ However, to our knowledge, SII and PMI, which are of interest as new inflammatory markers used in infectious diseases in children and adults, have not been examined in brucellosis cases.^13,14^ Although we consider that the STAT is sufficient for diagnosing brucellosis, we aimed to investigate the diagnostic value of indirect inflammation markers NLR, SII, and PMI in children with brucellosis. For this purpose, it was investigated whether inflammation markers differ between brucellosis pediatric cases and control groups and whether inflammation markers differ in brucellosis cases with and without focal involvement.

## METHOD

This study included 71 children diagnosed with brucellosis in the pediatric clinic of a tertiary hospital between January 1, 2017 and January 30, 2023. Two patients were excluded because they did not meet the diagnostic criteria. A control group consisting of 69 age- and gender-matched healthy children was determined. Cases were identified by retrospective review of hospital records according to the ninth revision of the International Classification of Diseases (ICD 9; A23.0-A23.9). Inclusion criteria were determined as being under 18 years of age and newly diagnosed with acute brucellosis. The diagnosis was made in patients with suggestive symptoms when the STAT titer was positive at 1:160 and above and/or *Brucella* species were isolated in blood culture. Patients who were under treatment or had recurrent attacks of brucellosis, with chronic brucellosis, had other accompanying acute or chronic diseases at the time of admission, or were taking medications were excluded. When a specific organ involvement was detected, the disease was defined as the focal form of brucellosis.^3^ The healthy group was composed of children who applied for health screening or evaluation before minor surgery (such as umbilical hernia, circumcision).

Permission to carry out the study was approved by the local Ethics Committee of Afyonkarahisar Health Sciences University (06.01.2023, 2023/1). All study procedures were performed following the Declaration of Helsinki.

Demographic, clinical, laboratory, and radiological imaging findings were examined from the patients’ electronic file records. Age, gender, complaint at presentation, duration of complaint, infection history (consumption of raw milk and dairy products, whether there was brucellosis in the family, whether the family was engaged in animal husbandry), and physical examination findings were recorded. From the laboratory data, complete blood count, C-reactive protein (CRP), erythrocyte sedimentation rate, alanine aminotransferase, aspartate aminotransferase, *Brucella* agglutination test, and blood culture results were examined. If performed, the findings of the patients who underwent abdominal, joint, and cranial imaging examinations and echocardiography were recorded.

White blood cell count, hemoglobin, neutrophil count, lymphocyte count, platelet count, and mean platelet volume (MPV) of both the case and control groups were noted. Anemia was defined as hemoglobin level below −2 standard deviations according to age and gender; leukopenia as white blood cell count <4500/μL; and thrombocytopenia was defined as a platelet count below 150,000/μL. NLR (ratio of neutrophil count to lymphocyte count), SII (platelet count x neutrophil count /lymphocyte count), PMI (platelet count × MPV/1000) were calculated as inflammation markers from the complete blood count. It was examined whether inflammation markers differed between groups and in brucellosis cases with and without focal involvement.

Statistical evaluation was performed using the Statistical Package for Social Sciences (SPSS) program for Windows, version 22.0 (Armonk, NY: IBM Corp.). Data were analyzed using descriptive statistical methods. Normality tests, including Kolmogorov-Smirnov and Shapiro-Wilk tests, were conducted to determine the distribution of quantitative data. Normally distributed data were expressed as mean±standard deviation, and non-normally distributed data were calculated as median and interquartile range. Categorical variables were expressed as percentage (%) and number (n). When comparing quantitative data between groups, Independent Samples *t* Test and analysis of variance (ANOVA) were used if the distribution was normal, and Mann-Whitney U test and Kruskal-Wallis test were used if the distribution was not normal. Chi-square and Fisher tests were applied for categorical variables. Receiver Operating Characteristic (ROC) curve analysis was employed for calculating the optimal cutoff values, sensitivity, and specificity of SII and PMI. Values of p<0.05 were considered statistically significant.

## RESULTS

The mean age of the 69 patients diagnosed with brucellosis included in the study was 11.9±3.7 years, and 69.5% (n=48) were male. The mean age of the healthy group was 10.4±2.6 years and 57.9% (n=40) were male. No significant difference was detected in terms of age and gender between the groups (p>0.05). In the case group, the most common complaints at admission were joint pain (75.3%) and fatigue (55%). The most commonly involved joints were the hip joint (27.5%) and the knee (17.3%). Of the 24 patients who presented with fever, 16 were acute and 8 were of unknown origin. The mean duration of complaints was 11.2±3.5 days. While 57.9% (n=40) of the patients had no findings on physical examination upon admission, 28.9% had arthritis, 8.6% (n=6) had hepatomegaly, and 4.3% (n=3) had hepatosplenomegaly. Of the total cases, 44.9% (n=31) were considered complicated due to arthritis, hepatitis, pancytopenia, and epididymitis. Demographic and clinical characteristics of the cases are shown in Table 1.

**Table 1:** Demographic and clinical characteristics.

Characteristic		Mean ± SD, median rangeor number (%) (n=69)
Mean age (year)		11.9±3.7 (1-17)
Gender		
	Female	21 (30.4)
	Male	48 (69.6)
Symptoms and clinical findings		
	Fever	24 (34.7)
	Arthralgia	52 (75.3)
	Fatigue	38 (55.1)
	Night sweating	14 (20.3)
	Weight loss	7 (10.1)
	Abdominal pain	7 (10.1)
	Headache	1 (1.4)
Source of contamination		
	Unpasteurized milk and dairy products	4 (5.7)
	Brucellosis in family	10 (14.4)
	Rural and farming	19 (27.5)
Focal involvement		
	Arthritis	20 (28.9)
	Hepatitis	7 (10.1)
	Pancytopenia	2 (2.8)
	Epididymitis	2 (2.8)
Joint involvement		
	Hip	19 (27.5)
	Knee	12 (17.3)
	Lumbosakral	6 (8.6)
	Ankle	3 (4.3)
	Wrist	1 (1.4)
	Shoulder	2 (2.8)
Serum agglutination test		
	1/160	32 (46.3)
	1/320	21 (30.4)
	≥1/640	16 (23.1)
Blood culture		
	Positive	18 (37.5)
	Negative	30 (62.5)
Treatment		
	Rifampicin + Tetracycline	19 (27.5)
	Rifampicin + Tetracycline + Gentamicin	46 (66.7)
	Rifampicin + TMP-SMX + Gentamicin	4 (5.8)

When hematological parameters were examined, anemia was detected in 4 (5.7%) cases, leukopenia in 10 (14.4%), leukocytosis in 2 (2.8%), thrombocytopenia in 5 (7.2%), and pancytopenia in 2 (2.8%) cases. The mean white blood cell count was 6,800/mm³ and there was no significant difference with the healthy group. Compared to the healthy group, hemoglobin and platelet values were significantly lower (p<0.001) and CRP and ferritin were significantly higher (p<0.001). SII and PMI were found to be significantly lower in the brucellosis patient group compared to the healthy group (p<0.001) (Table 2). In the comparison of cases with and without focal involvement, no statistically significant difference was detected in hematological parameters and inflammation markers (Table 3).

**Table 2:** Comparison of hematologic parameters between brucellosis group and control group.

Characteristics	Brusellosis group(n=69)	Healty group(n=69)	p-value
WBC (x10³/L)	6.8 (±3.2)*	6.9 (±2.3)*	0.137
Neutrophils (x10³/L)	2.9 (±2.2)*	3.4 (±1.4)*	0.057
Lymphocytes	2.9 (2.6)*	2.6 (±0.8)*	0.667
Platelets (x10^9^/L)	248±74*	313 (±107)*	<**0.001**
NLR	1.3(±0.8)*	1.3(±0.9)*	0.054
SII(x10^6^/L)	280 (209)†	432(282)†	<**0.001**
PMİ	2300 (84)†	2976 (±106)†	<**0.001**
MPV fL	9.6 (±1.3)*	10 (±1.0)*	0.043
Hgb g/dL	12.7±1.4*	13.7 (±1.1)*	<**0.001**
Ferritin μg/L)	225 (315)†	40 (±10.4)	<**0.001**
CRP (mg/L)	2.3 (5.2)†	0.1(0.1)†	<**0.001**

WBC: white blood cell; NLR: neutrophil-to-lymphocyte ratio; SII: systemic immune-inflammation index; PMİ: platelet mass index. MPV: mean platelet volüme, Hgb: hemoglobin, CRP: C-reactive protein.

Parameters were expressed as n (%). Bold indicates statistically significant p-values.

* mean±standard deviation;

† median (interquartile range).

**Table 3:** Comparison of inflammation markers based on focal involvement in brusellosis.

Characteristics	Brucellosis focal involvement positive(n=30)	Brucellosis focal involvement negative (n=39)	p-value
WBC (x10³/L)	6.8 (±3.4)*	5.7 (2.8) ^+^	0.204
Neutrophils (x10³/L)	3.5 (±3.0)*	3.4 (1.4) ^+^	0.306
Lymphocytes	2.8 (1.2)†	2.5 (1.5) ^+^	0.453
Platelets (x10^9^/L)	244±72*	252 (76) ^+^	0.665
NLR	1.3 (±1.0)*	1.0 (0.64) ^+^	0.268
SII(x10^6^/L)	282 (±271)*	269 (±196)*	0.790
PMİ	2338 (±91)*	2489 (±62)*	0.305
MPV fL	9.5 (±1.0)*	9.7 (0.9) ^+^	0.490
Hgb g/dL	12.6±1.2*	12.7 (±1.6)*	0.772
Ferritin μg/L)	225 (365)†	227 (312) ^+^	0.859
CRP (mg/L)	2.4 (5.6)†	1.6 (4.4)*	0.385

WBC: white blood cell; NLR: neutrophil-to-lymphocyte ratio; SII: systemic immune-inflammation index; PMİ: platelet mass index, MPV: mean platelet volüme, Hgb: hemoglobin, CRP: C-reactive protein.

Parameters were expressed as n (%).

* mean±standard deviation;

† median (interquartile ran).

**Figure 1:**
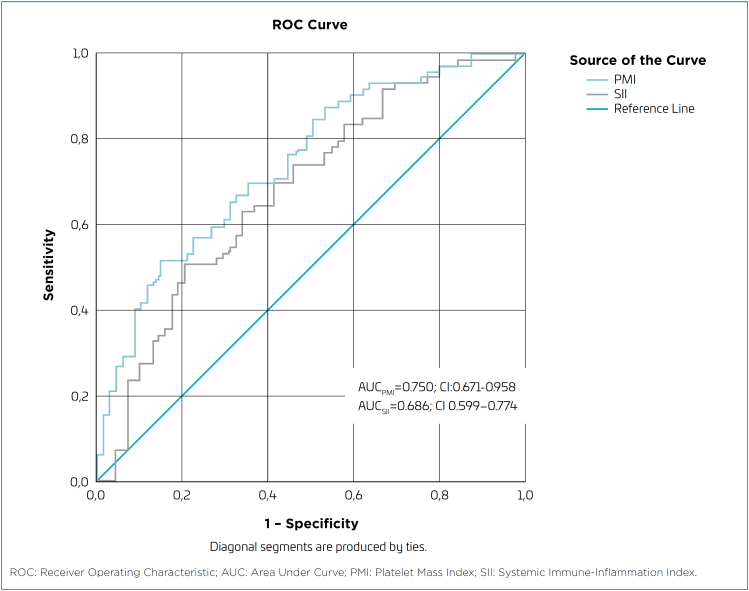
Receiver operating characteristics curve of platelet mass index and systemic immune-inflammation index to distinguish cases and control groups.

According to the ROC curve analysis in differentiating the case and control groups, the optimal cutoff value for PMI was found to be 2,557 (70% sensitivity; 65% specificity; area under the ROC curve [AUC] 0.750; confidence interval [CI] 0.671–0.958) and for SII, it was found to be 311 (70% sensitivity; 60% specificity; AUC 0.686; CI 0.599–0.774) (Figure 1).

## DISCUSSION

This is the first study to evaluate PMI in brucellosis and it was found to be significantly lower than control, with higher sensitivity and specificity than other parameters. PMI showed to be more predictive than platelet count and MPV alone.

It is known that platelets, in addition to their regulatory effects on the immune system, also play an active role in inflammation.^12^ Since activated platelets release a large number of substances that are important mediators of inflammation,^15^ the relationship of markers reflecting activation of platelets with the severity of inflammation in various diseases has been a matter of curiosity. For example, MPV, a marker of the average size of platelets, has recently been investigated as an indicator of both platelet activation and inflammation severity.^16^ MPV reflects platelet size and platelet production rate in the bone marrow, and many studies have shown an inverse relationship between MPV and platelet count in critically ill patients.^17^ Although high MPV has been attributed to larger platelets resulting from increased turnover of reactive platelets and has been reported to reflect platelet activation and inflammation severity,^18^ the results of studies examining the relationship between MPV and inflammatory processes in the literature are conflicting. While some studies show that MPV increases as the inflammation severity increases, other studies show that it decreases or does not change.^19,20^ Recent studies have also revealed that thrombocytopenia in brucellosis is accompanied by a decrease in MPV.^21,22^ However, in the present study, no significant difference was detected between the brucellosis case group and the control group concerning MPV. Supporting the contradictions in the literature, the use of MPV as an inflammation marker in brucellosis cases carries a question mark.

On the contrary, this study indicates that PMI was significantly lower in brucellosis cases. It is known that under physiological conditions, MPV is inversely proportional to the number of platelets to maintain hemostasis and a constant platelet mass, but this physiological ratio is disrupted in various pathologies.^23^ Recently, it has been demonstrated that platelet functions are better defined with PMI as a new marker.^24^ In previous studies, it was found to be significant as a prognostic factor in sepsis and transient tachypnea of the newborn in children and in Fournier's gangrene in adults.^13,25,26^ According to the findings of this study, PMI may be a more useful marker than platelet count or MPV alone. The results revealed that although PMI is supported as a diagnostic marker in cases of brucellosis, PMI does not differ between brucellosis cases with and without focal involvement. This may be because the severity of inflammation does not change with focal involvement. The fact that there is no difference in leukocyte count and CRP values between the two groups is consistent with our opinion.

NLR has been investigated in many diseases as a practical indicator of systemic inflammatory status and as a new marker reflecting the severity of systemic inflammation.^6,27^ Studies have shown that NLR is increased in many conditions, such as infectious diseases like pneumonia and appendicitis, and non-infectious diseases like febrile convulsions and obesity.^7,8,28,29^ In the study conducted by Cursi et al. in 649 children, they reported that NLR increased in patients with tuberculosis, which could support the diagnosis in case of clinical suspicion.^30^ Although there has been indication that increased NLR values in childhood brucellosis can be used as an indicator of inflammation and that NLR may reach abnormally high levels in children with brucellosis arthritis, Kazanasmaz et al.^31^ found no significant difference in median NLR values between brucellosis positive and negative groups. Sen et al.^32^ also did not find significant NLR in terms of predicting complicated brucellosis.^21,31-33^ In the present study, no significant difference was detected when comparing the NLR in the case group with the control group. No change was observed in NLR, either in the control group or in the presence of focal involvement. These results show that more data are needed on NLR values in the differential diagnosis of childhood brucellosis or in predicting focal involvement.

This study is also the first to examine SII, another current marker in childhood brucellosis. Studies in adults have shown that SII is associated with poor prognosis and increased mortality in cardiovascular disease, malignancies, and COVID-19.^11,14^ Studies in children are quite limited. Güngör et al. evaluated SII in cases with pleural effusion and empyema, and Kart et al. assessed SII in cases of perforated and acute appendicitis.^9,34^ In these studies, it was stated that SII, which increases as a marker of inflammation, also increases according to clinical severity. It has been reported that the SII value increases in COVID-19 and multisystem inflammatory syndrome in children (MIS-C) cases but does not change according to clinical severity. In this study, unlike in the literature, SII was found to be significantly lower in brucellosis cases than in the control group. This finding may be related to the more frequent occurrence of cytopenia in acute brucellosis. *Brucella* bacteria infect both peripheral (spleen) and central (bone marrow) organs of the reticuloendothelial system, leading to cytopenia due to bone marrow suppression or destruction of various blood cells. Additionally, it is known that hematological complications such as anemia, thrombocytopenia, and leukopenia are more common in acute brucellosis cases, unlike chronic and recurrent cases.^35^ It can be said that more comprehensive studies are needed before SII can be used as an inflammatory marker.

The first limitation of this study is the small sample size since it is a single-center study. Its second limitation is that it is performed on patients with different focal involvement. So that this may carry the risk of affecting hematological markers in a variable manner. More clear results can be obtained if cases with single tissue involvement (for example, only arthritis or only hepatitis) are investigated in future studies. The strength of this study is that it is the first study to examine PMI and SII markers in childhood brucellosis. Since the number of studies investigating practical hematological markers in brucellosis is limited, it also contributes to the literature with its findings.

In conclusion, this study showed that PMI, one of the new markers that can be calculated from complete blood count, a cheap and easily applicable laboratory test, may be a useful marker in the diagnosis of childhood brucellosis. It also supports the view that PMI is a better marker of inflammatory processes than platelet count or MPV alone. On the other hand, since the results obtained from this study are incompatible with the literature, the diagnostic guidance of NLR or SII in brucellosis cases appears to be insufficient. New studies are needed on the usefulness of PMI, NLR, and SII in the diagnosis process of childhood brucellosis.

## Data Availability

The database that originated the article is available with the corresponding author.
